# Role of Lipotoxicity and Contribution of the Renin-Angiotensin System in the Development of Polycystic Ovary Syndrome

**DOI:** 10.1155/2018/4315413

**Published:** 2018-06-03

**Authors:** Alexandre Connolly, Samuel Leblanc, Jean-Patrice Baillargeon

**Affiliations:** ^1^Department of Pharmacology-Physiology, Faculty of Medicine and Health Sciences, 3001 12e Avenue Nord, Université de Sherbrooke, Sherbrooke, QC, Canada J1H 5N4; ^2^Division of Endocrinology, Department of Medicine, Faculty of Medicine and Health Sciences, 3001 12e Avenue Nord, Université de Sherbrooke, Sherbrooke, QC, Canada J1H 5N4

## Abstract

Polycystic ovary syndrome (PCOS) is a common and significant condition associated with hyperandrogenism, infertility, low quality of life, and metabolic comorbidities. One possible explanation of PCOS development is cellular dysfunction induced by nonesterified fatty acids (NEFAs), that is, lipotoxicity, which could explain both the hyperandrogenemia and insulin resistance that characterize women with PCOS. The literature suggests that androgen biosynthesis may be induced by overexposure of androgen-secreting tissues to NEFA and/or defective NEFA metabolism, leading to lipotoxic effects. Indeed, lipotoxicity could trigger androgenic hyperresponsiveness to insulin, LH, and ACTH. In most PCOS women, lipotoxicity also causes insulin resistance, inducing compensatory hyperinsulinemia, and may thus further increase hyperandrogenemia. Many approaches aimed at insulin sensitization also reduce lipotoxicity and have been shown to treat PCOS hyperandrogenemia. Furthermore, our group and others found that angiotensin II type 2 receptor (AT2R) activation is able to improve lipotoxicity. We provided evidence, using C21/M24, that AT2R activation improves adipocytes' size and insulin sensitivity in an insulin-resistant rat model, as well as androgen levels in a PCOS obese rat model. Taken together, these findings point toward the important role of lipotoxicity in PCOS development and of the RAS system as a new target for the treatment of PCOS.

## 1. Introduction

Polycystic ovary syndrome (PCOS) is a common condition affecting 6–8% of women of childbearing age [1]. It is the most frequent endocrine disorder among young women in North America. In addition to being the most frequent cause of female anovulatory infertility [2], PCOS is the commonest cause of hyperandrogenism in women [3], thus leading to esthetical concerns such as excessive hair growth, acne, and male-pattern alopecia. In addition, PCOS is currently considered a paradigm of cardiometabolic disease, since the prevalence of metabolic syndrome [4], dyslipidemia [5], and type 2 diabetes [6, 7] is much higher in PCOS women than in normal age- and BMI-matched women. Indeed, PCOS women display metabolic insulin resistance and compensatory hyperinsulinemia [8], which play a critical role in the syndrome's development [9, 10]. Insulin resistance is classically defined as the reduced ability of insulin to stimulate glucose disappearance in peripheral tissues as well as inhibit hepatic glucose production and adipose tissue lipolysis [11, 12], which corresponds to the main metabolic actions of insulin.

Lipotoxicity, referring to the cellular adverse consequences of nonesterified fatty acids (NEFAs), is widely studied for its implication in the development of dysfunctions in adipose tissue, muscle, and liver and in pancreatic *β*-cells [13, 14], leading to metabolic insulin resistance and type 2 diabetes. Lipotoxicity could also be implicated in PCOS pathophysiology, both through increased androgen production and through induction of systemic insulin resistance leading to hyperinsulinemia. Yet, many questions remain unanswered regarding the mechanisms by which metabolic insulin resistance and/or lipotoxicity leads to hyperandrogenemia and how this could be prevented or reversed.

To control the serious short- and long-term consequences of PCOS, it is becoming primordial to identify new therapeutic targets in the treatment of PCOS. To this day, the first line of treatment for infertility and hyperandrogenism in PCOS women is lifestyle management [15], which is difficult to maintain during the long term and probably ineffective in most normal-weight women. The main pharmacologic treatment recommended for PCOS, that is, hormonal contraception, is effective to control PCOS symptoms but not cardiometabolic risks, and other drugs, such as the insulin sensitizer metformin, are too weak to fully treat PCOS manifestations in most women. However, recent evidence suggests a potential benefit of type 2 angiotensin II (Ang II) receptor (AT2R) stimulation for improving both lipotoxicity and hyperandrogenism. Although we recognize that other important mechanisms and effective therapeutic approaches are of interest in PCOS, this review will focus on the role of lipotoxicity as an innovative hypothesis for PCOS development and of the AT2R pathway as a new target for the management of women with PCOS.

## 2. Role of Lipotoxicity and Insulin Action in the Development of Polycystic Ovary Syndrome

### 2.1. Insulin Actions and Metabolic Insulin Resistance

**Figure 1:**
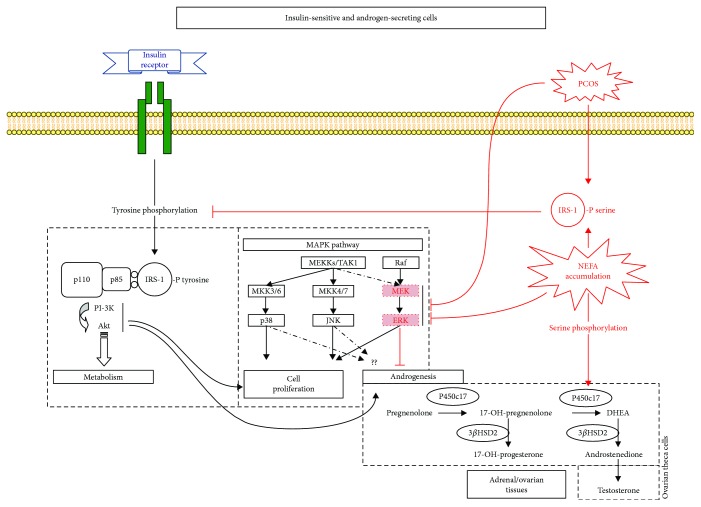
Proposed cellular mechanisms involved in insulin-stimulated androgen biosynthesis, PCOS-associated defects, and free fatty acid-induced insulin resistance and androgen production, together with AT2R and PPAR*γ* actions. Serine phosphorylation of IRS-1 prevents its binding with PI3K and inhibits insulin signaling. Moreover, serine phosphorylation of P450c17 increases its 17,20-lyase activity and thus androgen biosynthesis. Interestingly, serine phosphorylation of IRS-1 is constitutively increased in PCOS women and increased by nonesterified fatty acid (NEFA) overload. Insulin-stimulated androgen production has been shown to be reduced by specific inhibition of PI3K and increased by specific inhibition of MEK. MEK/ERK activity was found to be constitutively reduced in PCOS women and inhibited by NEFAs. It was also suggested that P450c17 activity may be stimulated by other players of the MAPK pathway, such as MKK3/6-p38 and MKK4/7-JNK, whose activities are not reduced, and may be even increased, in women with PCOS. Adapted from [10].

Despite variations caused by fasting and food intake, plasmatic glucose levels remain stable due to the metabolic actions of insulin on target tissues, such as increasing glucose uptake by muscles and adipose tissues and inhibiting glucose production in the liver [16]. Insulin mediates its actions through a tyrosine kinase receptor, which is a heterotetrameric protein formed of two extracellular alpha subunits and two transmembrane beta subunits. Once activated by insulin binding, the insulin receptor changes its conformation, which increases its beta subunit kinase activity, leading to phosphorylation of its intracellular substrates. Two major types of actions can then be mediated, as illustrated in the left part of Figure 1: metabolic and mitogenic actions. For metabolic actions, members of the insulin receptor substrate (IRS) family are phosphorylated by the insulin receptor, which are then recognized by effector molecules, such as PI3-K that triggers the recruitment of Akt, an important signal transduction molecule for glucose uptake, regulation, and metabolism. For mitogenic actions, such as cell growth and differentiation, insulin receptors phosphorylate Shc or IRS, enabling their association with Grb2 and SOS, resulting in Ras activation that initiates a cascade of phosphorylations leading to MAPK/ERK activation [16].

In pathologic situations like obesity, regulation of insulin signaling is altered by numerous internal and external stimuli, including nutrients like lipids, leading to metabolic insulin resistance [13] and other consequences (discussed below). In this situation, impaired glucose uptake in insulin-sensitive tissues and hepatic glucose production lead to an increase in insulin secretion in order to maintain normal glucose levels, that is, compensatory hyperinsulinemia. This is possible as long as pancreatic *β*-cells can secrete enough insulin to compensate. If not, glucose levels will rise to levels compatible with diabetes.

### 2.2. Insulin Resistance in Polycystic Ovary Syndrome

Defective action of insulin in PCOS women has been well described and confirmed in many studies since more than 25 years [8]. Insulin sensitivity in PCOS women is 35 to 40% lower than that in healthy women with similar BMI [17–19], such that most PCOS women display insulin resistance and compensatory hyperinsulinemia, which seem to play an important role in PCOS pathogenesis [9, 10]. Using the gold standard method, the glucose-insulin clamp, to assess metabolic insulin resistance, many studies in different populations found that insulin sensitivity was significantly lower among obese and nonobese PCOS women in comparison to healthy women within the same obesity subgroup [8, 11, 12, 20]. However, some studies in the subgroups of nonobese women found that insulin sensitivity was similar among women with or without PCOS [21, 22] or that the lower insulin sensitivity observed in PCOS women disappeared after correction for truncal-abdominal fat distribution [23]. Furthermore, Ciampelli et al. discovered that lean PCOS women with normal insulin levels had completely normal insulin sensitivity [24]. In summary, among nonobese women with PCOS, some display insulin resistance that may be due to abdominal obesity and others are not more insulin resistant than the general population.

Altogether, these results suggest that insulin resistance and hyperinsulinemia certainly contribute to either unveiling or worsening of the syndrome in women predisposed to PCOS, but it may not be the actual cause of the predisposition to the syndrome, at least in some women with PCOS. Selection of women who have a more severe PCOS phenotype due to the contribution of insulin resistance and/or hyperinsulinemia would explain why studies usually found that PCOS women display lower insulin sensitivity than controls on average, even within BMI subgroups.

### 2.3. Implication of Insulin Action in PCOS Hyperandrogenemia Based on Clinical Studies

*In vivo* studies have shown that women with PCOS display both adrenal and ovarian androgenic hyperresponsiveness to LH [25, 26] and ACTH [27, 28]. Interestingly, many studies found that this exaggerated androgenic response was curbed after treatment improving metabolic insulin resistance in both lean and obese PCOS women [28–31], but not after chronic suppression of LH [25, 26] or ACTH [32, 33]. These findings suggest that this hyperresponsiveness is not due to chronic LH or ACTH activation, but maybe caused by insulin resistance or related factors.

Regarding studies linking insulin and androgen production, Nestler et al. were among the first to show that insulin levels were directly related to androgen production *in vivo* [34]. They showed that reducing *β*-cell secretion of insulin in obese and hyperinsulinemic PCOS women with diazoxide, which reduces insulin secretion directly in pancreatic *β*-cells, lowered significantly free and total testosterone after only 10 days. A 6-month randomized control trial also demonstrated that lowering postprandial insulin levels, using acarbose that slows down intestinal glucose absorption, significantly decreases the free androgen index among obese hyperinsulinemic PCOS women, in comparison to the placebo [35].

Moreover, we showed in lean normoinsulinemic PCOS women who were not insulin resistant based on a glucose-insulin clamp that lowering their insulin secretion with diazoxide for 8 days was associated with a significant drop in their free testosterone and androstenedione levels [36]. Since their insulin levels were normal at the baseline, this study suggests that lean, insulin-sensitive women with PCOS display hyperresponsiveness to insulin, which could also be the case for all women with PCOS. Our group demonstrated as well that nonobese normoinsulinemic women with PCOS randomized to either metformin, rosiglitazone, or the combination of both significantly lowered their free testosterone levels in comparison to the placebo group [37]. In this study, rosiglitazone, a PPAR*γ* agonist improving lipotoxicity and insulin sensitivity, reduced androgen levels without any effect on insulin levels. This result suggests that PPAR*γ* agonists may target the mechanisms by which insulin exaggeratedly stimulates androgen production in women with PCOS, thus restoring normal androgenic response to insulin.

It is important to mention that, in healthy women, inhibition of insulin secretion with diazoxide does not have any effect on their testosterone levels [38] and hyperinsulinemia during a euglycemic-hyperinsulinemic clamp does not increase their androgen production [39]. Accordingly, insulin does not seem to be related to androgen production in normal women, suggesting that androgenic hyperresponsiveness to insulin is specific to PCOS [9]. Taken together, these results suggest that insulin contributes to PCOS hyperandrogenemia even when insulin levels are normal and that women may develop PCOS because of androgenic hyperresponsiveness not only to ACTH and LH but also to insulin [9, 10]. This is important because insulin levels can increase many folds with insulin resistance, while LH and ACTH levels are more stable.

Insulin also contributes to hyperandrogenemia in PCOS women by lowering the liver production of sex hormone-binding globulin (SHBG), which acts as a testosterone carrier in plasma and thus lowers free testosterone levels. Circulating SHBG levels are indeed lower in obese, hyperinsulinemic women with PCOS [40]. These low levels of SHBG were improved in obese women with PCOS by treatment with diazoxide for 10 days or acarbose for 6 months [35, 41]. Low SHBG levels are also found in insulin-resistant patients with prediabetes or type 2 diabetes [40].

### 2.4. Insulin Signaling Pathways in Women with PCOS (Figure 1)

Most studies in women with PCOS were performed on insulin-sensitive tissues, such as muscle and adipose tissues, and also on fibroblasts that are easily accessible. In fibroblast [42] and muscle cells [43], it was found that serine phosphorylation of the insulin receptor or insulin receptor substrate- (IRS-) 1 is constitutively increased in PCOS women (see Figure 1, in red), which was associated with a reduction in tyrosine phosphorylation of the insulin receptor and IRS [42] and reduced PI3K activity. It was indeed clearly demonstrated in type 2 diabetes that signal transduction of the metabolic pathway of insulin is reduced when insulin receptors and IRS-1 or IRS-2 are serine-phosphorylated instead of tyrosine-phosphorylated [16, 44]. Accordingly, a study performed in muscular biopsies obtained during *in vivo* insulin infusion in obese PCOS women found that the association between IRS-1 and PI3K was altered, which was correlated with reduced *in vivo* glucose uptake [19]. Defective metabolic actions of insulin in PCOS women seem to involve initial insulin signaling steps and most likely result from abnormal serine phosphorylation rather than from defective protein expression.

Insulin mitogenic actions seem to be affected differently than metabolic actions in women with PCOS. In fibroblasts cultured from PCOS women, as compared to healthy controls, glucose uptake and glycogen synthesis were reduced, but no difference was seen in cell proliferation under insulin or IGF-1 stimulation [18]. Another study in skeletal muscle from PCOS women found that insulin-mediated activation of ERK1/2, an important mediator of the mitogenic pathway, was severely attenuated during an insulin-glucose clamp, as compared to control women [45] (see Figure 1, in red).

### 2.5. Implication of Insulin Signaling Pathways in Androgen Biosynthesis (Figure 1)

Ovarian androgens are produced by theca cells, which surround granulosa cells, and by interstitial cells, which are scattered between follicles. Theca-interstitial cells express the enzyme P450c17 and are stimulated by LH (or hCG). Granulosa cells however, which are juxtaposed to theca cells and surround the oocyte, do not express P450c17 and convert theca-interstitial cell-derived androgens to estrogens under FSH stimulation. In women with PCOS, hyperandrogenemia is driven by high P450c17 and 3*β*HSD activities [46], the key enzymes of androgen biosynthesis. As illustrated in the right part of Figure 1, P450c17 has both 17*α*-hydroxylase and 17,20-lyase activities [47–49], the latter generating the main androgen precursors, dehydroepiandrosterone (DHEA) and androstenedione. This lyase activity is favored by different factors, including serine/threonine phosphorylation of P450c17 [50]. Since P450c17 has a significant role in androgen production, any change in its expression or activity will therefore have an important impact on androgen production.

The cellular mechanisms by which insulin regulates androgen production are not well understood, but potential pathways are illustrated in Figure 1 [10]. Studies have shown that insulin potentiates androgen production in normal ovarian cell models [10], and even more so in cultured ovarian cells from PCOS women, through direct binding to its own receptor [51, 52]. Specific blockade of PI3K in normal human theca cells markedly inhibits the combined insulin and LH stimulation of P450c17 activity [53]. Insulin may also stimulate P450c17 activity through some members of the MAPK pathway, such as p38 and JNK (for review, see [54] and Figure 1). Importantly, these pathways appear to be enhanced in women with PCOS [55, 56], as opposed to the PI3K pathway that was shown to be insulin resistant [18, 19, 55, 57, 58]. Furthermore, several findings suggest that a reduction of ERK1/2 signaling, another component of the MAPK insulin pathway, may increase androgen biosynthesis [53, 59, 60] and be intrinsic to PCOS women [59]. Thus, the positive balance between p38/JNK/p54 and ERK insulin pathways seems to be characteristic of PCOS and to upregulate P450c17 activity and could therefore explain PCOS androgenic hyperresponsiveness. Interestingly, cellular overexposure to nonesterified fatty acid (NEFA), which leads to lipotoxicity, has been shown to inhibit ERK activity in other cell models and could therefore mimic this defect.

### 2.6. Lipotoxicity, Metabolic Insulin Resistance, and PCOS

Lipotoxicity refers to the cellular adverse consequences of NEFA [44]. Cellular NEFAs originate either from circulating NEFA or from triglycerides carried in the circulation by triglyceride-rich lipoproteins (chylomicrons and VLDL), which release NEFA under the action of lipoprotein lipase. Normal intracellular NEFA metabolism could be exceeded or impaired, as previously described in association with a genetic defect in mitochondrial *β*-oxidation in offspring of subjects with type 2 diabetes [61]. Our group has demonstrated *in vivo* that prolonged elevation of circulating NEFA reduces muscle and hepatic insulin sensitivity, as well as *β*-cell function, in normal subjects [38, 62–64]. This is one likely mechanism by which lipotoxicity can lead to global metabolic insulin resistance and diabetes [65]. Recent studies found that increasing circulating NEFA levels, using an infusion of intralipid and heparin, stimulates androgen production in healthy men [66] and women [67], suggesting that systemic NEFA overflow can also trigger androgen production *in vivo*.

Regarding intracellular mechanisms of lipotoxicity, it was shown that mitochondrial NEFA *β*-oxidation overload may lead to the formation of reactive lipids, such as diacylglycerol (DAG) and ceramides (for review, see [68]). These mediators activate the serine/threonine kinase cascade leading to serine phosphorylation of the insulin receptor and insulin receptor substrates (IRS-1/2), causing a decrease in PI3K activation [14, 69–73] (Figure 1). As mentioned above, constitutively increased serine phosphorylation levels of insulin receptors and IRS-1 have been found in PCOS skin fibroblasts [42, 74] and muscle cells [19, 43, 75]. Most importantly, it was shown that serine phosphorylation of P450c17 increases its 17,20-lyase activity [50] (Figure 1). Moreover, we demonstrated in bovine adrenal cells that, under LH pathway activation, the saturated fatty acid palmitate increases androgen production, concomitantly with a decrease in ERK1/2 phosphorylation [76], as previously observed in PCOS theca cells [59]. Thus, we demonstrated that lipotoxicity can directly trigger androgen overproduction *in vitro*, with inhibition of the MEK/ERK pathway as a potential mechanism. Furthermore, using follicular fluid as a surrogate of the intraovarian milieu, we showed that local gonadotropin-induced androgen production is associated with intraovarian exposure to lipids and that this association may be mediated by ineffective NEFA *β*-oxidation (Figure 1, bottom left part) [77].

In this study, we also found that the inflammation was associated with androgen production as well, although to a lesser extent (reflected by IL-6) [77]. As for NEFA metabolites, the inflammation marker TNF-*α* is known to serine-phosphorylate IRS-1 [78], thus contributing to insulin resistance. Indeed, systemic or low-grade tissue inflammation has been implicated in the development of metabolic disorders including type 2 diabetes [78]. On the other hand, inflammation may be triggered by lipotoxic effects [14, 79]. Inflammation is therefore a potential mechanism for PCOS development, directly or as a mediator of lipotoxicity. The discussion of the role of inflammation in PCOS is beyond the scope of this review, and the reader is referred to a recent review from González [80].

**Figure 2:**
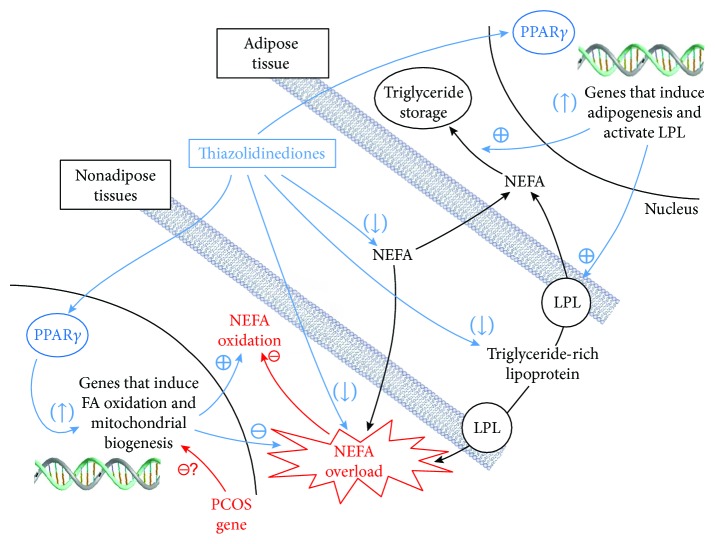
PPAR*γ* and AT2R implications in PCOS-associated defects. PPAR*γ* (and maybe AT2R) agonists increase tyrosine phosphorylation of IRS-1. Insulin-stimulated androgen production has been shown to be increased by specific inhibition of MEK. MEK/ERK activity was found to be constitutively reduced in PCOS women, inhibited by NEFAs, and activated by PPAR*γ* agonists. AT2R activation counteracts lipotoxic effects of NEFA either directly or through activation of PPAR*γ*. Adapted from [10].

While inducing lipotoxicity was shown to trigger androgen production *in vivo* and *in vitro*, improving lipotoxicity through PPAR*γ* activation was also found to reduce androgen levels in women with PCOS [81]. *In vivo* studies showed that PPAR*γ* agonists reduce PCOS androgenic hyperresponsiveness to LH [25, 26] and ACTH [28, 32, 33, 82–85] and probably also to insulin [37]. As illustrated in Figure 2, activation of PPAR*γ* improves lipotoxicity by two main mechanisms: (1) upregulation of key genes involved in NEFA and triglyceride storage in adipose tissue [86, 87]. By promoting triglyceride storage in adipose tissues, PPAR*γ* agonists reduce circulating NEFA [88] and thus prevent NEFA overexposure of nonadipose tissues [89]. (2) Upregulation of genes that are important for mitochondrial biogenesis and NEFA *β*-oxidation in nonadipose tissues [90]. In PCOS women, expression of these genes was reduced in skeletal muscle as compared to healthy women, which was associated with insulin resistance, and their expression was upregulated after treatment with a PPAR*γ* agonist [91]. Furthermore, it was found that PPAR*γ* is present in ovarian theca cells and that its activation impedes both P450c17 activities and LH- and/or insulin-stimulated testosterone production [92–94]. PPAR*γ* agonists have also been shown to reverse the enhanced expression of P450c17 induced by specific inhibition of MEK/ERK [60] (Figure 1). Thus, PPAR*γ* seems implicated in androgen biosynthesis, and its activation may improve the balance between p38/JNK and ERK pathways associated with PCOS androgenic hyperresponsiveness.

## 3. The Angiotensin II Type 2 Receptor: A New Target for the Management of Polycystic Ovary Syndrome

### 3.1. The Renin-Angiotensin System and Angiotensin II Receptors

Several recent reviews, including ours, clearly implicate the renin-angiotensin system (RAS) in the development of insulin resistance, type 2 diabetes, and cardiovascular complications [95]. The RAS is mainly known for its role in regulating blood pressure. Indeed, when the juxtaglomerular cells in the kidney detect a drop in the circulating volume of plasmatic sodium levels, they secrete renin. This enzyme then cleaves the dodecapeptide angiotensinogen (produced by the liver) into decapeptide angiotensin I. Angiotensin I is then cleaved by the angiotensin-converting enzyme (ACE1), a carboxypeptidase which is located on the endothelial cells of capillaries, to form octapeptide angiotensin II [96]. Classically, angiotensin II (Ang II) mediates its action via the Ang II type 1 (AT1R) and type 2 (AT2R) receptors.

AT1R is expressed almost ubiquitously in the adult and is well known for being important in maintaining blood pressure and hydromineral balance [97]. Several studies have shown that chronic activation of the AT1R may contribute to insulin resistance and metabolic disorders in positive energy balance conditions [98–100]. Part of this action is mediated by AT1R-induced serine phosphorylation of the insulin receptor and IRS-1, thus inhibiting the downstream cascade of insulin signaling. Many reports have shown that AT2R activation reverses the negative action of AT1R on insulin receptor signaling (reviews: [101, 102]) and this is one of the mechanisms by which AT2R might exert its beneficial effects on insulin resistance.

In contrast to AT1R, AT2R expression is low in most tissues [103], except for steroidogenic tissues such as adrenal glands and ovaries [104, 105]. However, it may be reexpressed in various disease states, including type 2 diabetes [98, 106, 107]. In the ovaries, AT1R and AT2R are localized in both theca and granulosa cells, in varying ratios according to the species [108]. In the ovary, it has been shown that AT2R activation (but not AT1R) stimulates conversion of androgen to estrogen [109, 110]. Importantly, AT2R is known for inhibiting the excessive effects of AT1R in various experimental models [101, 102].

### 3.2. Benefits of Angiotensin II Type 2 Receptor Activation on Lipotoxicity and Androgen Production

Large clinical trials have provided evidence that AT1R blockers improve insulin sensitivity and help prevent type 2 diabetes [111, 112]. With regard to women with PCOS, a case series has shown that treatment of four obese and hypertensive PCOS women with the AT1R antagonist telmisartan caused a marked reduction in the androgen levels of all four women and improved menstrual cyclicity in three of the women [113]. These benefits were achieved despite a nonsignificant change of fasting insulin levels in these women who were not severely insulin resistant at the baseline. Moreover, a prospective observational study found that treatment of ten overweight/obese and hypertensive PCOS women with the angiotensin-converting enzyme inhibitor lisinopril for 4 weeks caused a significant decrease in their free testosterone levels with no change in SHBG levels [114]. A meta-analysis has also suggested that polymorphisms of the angiotensin-converting enzyme gene were associated with increased risk of PCOS in Caucasian women [115]. These findings suggest that the renin-angiotensin system may play a role not only in insulin resistance and type 2 diabetes but also in the development or management of PCOS.

Of note, the effects of blocking AT1R may result not only from the inhibition of AT1R but also from unopposed activation of AT2R [98, 116]. However, prior to 2004, the role and impact of AT2R stimulation had been difficult to establish due to the absence of a selective and potent AT2R agonist. Fortunately, we and others have now extensively characterized the properties of a nonpeptide drug-like compound, *C21/M24*, which is a highly selective AT2R agonist [117].

Several animal *in vivo* studies showed that AT2R activation with C21/M24 counteracts many deleterious effects of AT1R activation [118, 119]. More importantly, using C21/M24 and an AT2R-knockout (AT2R-KO) mouse model, our group put forward several lines of evidence supporting that the stimulation of AT2R could prevent lipotoxicity and its consequences. In primary cultures of rat preadipocytes, C21/M24 increased PPAR*γ* expression and favored cell differentiation [120], which are important for the storage of NEFA in adipose tissue and the prevention of NEFA spillover to nonadipose tissues. Accordingly, C21/M24 treatment improved insulin resistance induced by 6 weeks of high-fat-high-fructose (HFHF) diet in Wistar rats [120]. These important results were recently confirmed by another group using a mouse model of type 2 diabetes [100]. Furthermore, it was found that tissue NEFA uptake, measured with the ^18^F-FTHA tracer, was greater in AT2R-KO mice as compared to wild-type mice [121], including in adrenal glands (unpublished, mouse ovaries were too small to assess). AT2R-KO mice were also unable to increase the size of their adipocytes during HF-HF feeding, as compared to WT mice [121]. This finding reflects the inability of AT2R-KO mice to store lipids and may explain the increase of NEFA uptake by their nonadipose tissues. Moreover, our study [120] and others [122] suggest that AT2R stimulation increases PPAR*γ* expression and activation. This might be a mechanism by which AT2R improves adipose tissue function. Altogether, these results suggest a beneficial role for AT2R in preventing tissue NEFA overload and insulin resistance.

Since AT2R stimulation with C21/M24 was found to improve lipotoxicity, our group proposed that this compound could potentially treat the underlying cause of PCOS. Accordingly, female JCR:LA-*cp*/*cp* rats, an obese model of PCOS displaying hyperandrogenism, insulin resistance, and polycystic ovaries, were treated with C21/M24 or intraperitoneal saline implants [123]. After only 7 days, C21/M24 treatment improved slightly and nonsignificantly insulin and NEFA levels in PCOS/*cp* rats as compared to saline but was able to normalize testosterone levels and ovarian NEFA uptake (measured using the ^18^F-FTHA tracer). When combining both PCOS/*cp* rat groups, testosterone levels were positively correlated with NEFA levels and even more strongly with ovarian NEFA uptake. These results suggest that direct stimulation of AT2R with C21/M24 improves rapidly and predominantly ovarian NEFA uptake and androgen production in obese, insulin-resistant PCOS rats.

### 3.3. Angiotensin II Type 2 Receptor Signal Transduction Mechanisms (Figure 3)

Despite the great deal of interest recently generated by the AT2R and its nonpeptide agonist C12/M24, the precise mechanisms by which the AT2R conveys its signals are not yet completely understood. While the AT2R is a G protein-coupled receptor, it is considered having atypical transduction mechanisms. Indeed, the stimulation of AT2R does not affect classical second messengers, such as the modulation of cAMP or production of InsP_3_ or DAG [124, 125]. *In vitro* studies reported that the G*α*i protein could bind to and be activated by the AT2R, suggesting that it may be a signaling pathway for AT2R [126, 127]. Indeed, AT2R stimulation was shown to activate nitric oxide (NO) synthase, which is known to be induced by G*α*i, and to increase both NO and cGMP in a G*α*i protein-dependent manner. This pathway would be necessary for cell differentiation and migration mediated by the AT2R [128, 129].

**Figure 3:**
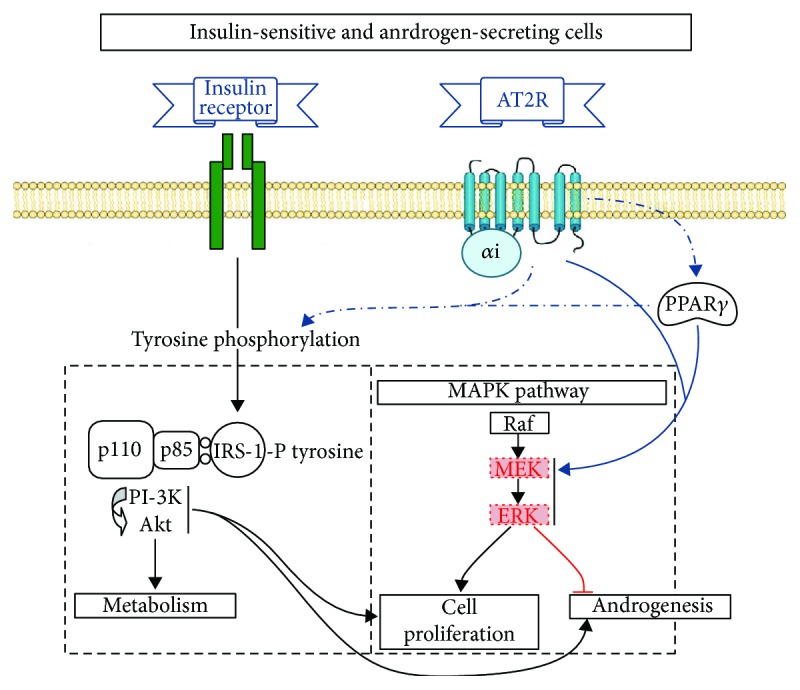
Whole-body and cellular mechanisms of PPAR effects. At the adipocyte level, activation of peroxisome proliferator-activated receptors (PPAR*γ*) increases the transcription of genes that induce adipogenesis as well as lipoprotein lipase (LPL) activation, which promotes the uptake of triglycerides by adipocytes. At the level of nonadipose tissues, activation of PPAR*γ* increases the transcription of genes that induce fatty acid (FA) oxidation and mitochondrial biogenesis, which decrease intracellular overload of FA and FA metabolites. A PPAR*γ*-induced reduction of circulating levels of nonesterified FA (NEFA) and triglyceride-rich lipoprotein contributes to the decrease in NEFA uptake by nonadipose tissues and the overload of NEFAs in these tissues.

Furthermore, depending on the model used, stimulation of the AT2R was shown to activate different tyrosine and serine/threonine phosphatases such as SHP-1, MKP-1, and PP2A, which can lead to transient inhibition of ERK1/2 and insulin signaling [130–136]. On the other hand, it was shown that AT2R can also induce sustained activation of ERK1/2 through the activation of Rap1/B-Raf [137, 138]. It is therefore possible that this sustained activation of ERK1/2 could counterbalance the inhibition of ERK1/2 that was observed in PCOS theca cells and that can be induced by NEFA (see Figures 1 and 3). Since constitutive inhibition of ERK1/2 was associated with higher androgen-secreting potential of theca cells (see previous sections and Figure 1), the ability of AT2R to activate ERK1/2 may play a role in correcting the NEFA-induced androgen hyperresponsiveness that characterizes women with PCOS (see Figure 3). Furthermore, it was shown in a cell line that AT2R stimulation is able to increase the expression of PPAR*γ* and its activity [122].

Taken together, these potential mechanisms of AT2R action indicate that this receptor could be very useful in correcting PCOS hyperandrogenism. Indeed, as illustrated in Figure 3, most of the factors that were identified as playing a role in the hyperandrogenism of women with PCOS, such as lipotoxicity, reduced MEK/ERK activity, and insulin resistance, could potentially be corrected by AT2R-specific activation.

## 4. Conclusion

In summary, we put forward in this review the hypothesis that NEFA-induced lipotoxicity may explain both the hyperandrogenemia and insulin resistance that characterize PCOS women. Indeed, the literature including our own contributions suggests that PCOS hyperandrogenemia may be due to overexposure of androgen-secreting tissues to NEFA and/or defective intracellular NEFA metabolism, leading to lipotoxic effects. Lipotoxicity could therefore be the cause of androgenic hyperresponsiveness to insulin, LH, and ACTH. Lipotoxicity is also well known to cause insulin resistance, which induces compensatory hyperinsulinemia and further increases hyperandrogenemia in many women with PCOS. On the other hand, treatments that reduce lipotoxicity, such as PPAR*γ* agonists, improve systemic and cellular metabolism of NEFA and have been shown to treat PCOS hyperandrogenemia. Furthermore, our group and others recently found that AT2R activation is also able to improve lipotoxicity. We discussed evidence showing that AT2R signaling improves adipocyte size and nonadipose tissue NEFA uptake and might therefore prevent lipotoxicity and insulin resistance. Altogether, this review of the literature pleads in favor of the development of new approaches or pharmacologic treatments targeting lipotoxicity, such as the newly developed agonist C21/M24, for the management of both short-term symptoms and long-term cardiometabolic consequences of PCOS.

## Additional Points

*Copyright and Permission*. To the best of our knowledge, the materials included in this chapter do not violate copyright laws. All original sources have been appropriately acknowledged and/or referenced. Where relevant, appropriate permissions have been obtained from the original copyright holders.
